# Formation and persistence of estragole-derived DNA adducts in human liver cells and tissue

**DOI:** 10.1007/s00204-026-04364-5

**Published:** 2026-03-31

**Authors:** G. Ackermann, M. Abel-Beckmann, C. Quarz, M. Halaczkiewicz, S. Stegmüller, E. Richling, G. Manolikakes, M. Christmann, J. H. Küpper, D. Schrenk, J. Fahrer

**Affiliations:** 1 Division of Food Chemistry and Toxicology, Department of Chemistry, Rheinland-Pfälzische Technische Universität (RPTU) Kaiserslautern-Landau, Erwin-Schrödinger-Str. 52, 67663 Kaiserslautern, Germany; 2 Institute of Toxicology, University Medical Center, Obere Zahlbacher Str. 67, 55131 Mainz, Germany; 3 Division of Organic Chemistry, Department of Chemistry, Rheinland-Pfälzische Technische Universität (RPTU) Kaiserslautern-Landau, Erwin‑Schrödinger‑Str. 54, 67663 Kaiserslautern, Germany; 4 Division of Molecular Cell Biology, Institute of Biotechnology, Brandenburg University of Technology Cottbus-Senftenberg, Universitätsplatz 1, 01968 Senftenberg, Germany

**Keywords:** Phenylpropenes, Estragole, Human liver tissue, Liver cells, Hepatotoxicity, Genotoxicity, DNA adducts, Repetitive exposure, Bitter fennel oil

## Abstract

Estragole (ES) is a phenylpropene naturally occurring in various herbs and spices, such as fennel and basil. Humans are exposed to ES via the diet or herbal medicinal products. Following its gastrointestinal resorption, hepatic bioactivation of ES by Cytochrome P450 monooxygenases (CYPs) and sulfotransferases (SULTs) can lead to DNA adduct formation and liver cancer in rodents. Up to now, no data is available on ES-dependent DNA adducts in human tissue, which would however be important for human risk assessment**.** Our work thus analyzed whether ES-derived DNA adducts are present in human liver samples. Furthermore, DNA adduct formation after repetitive ES exposure and upon incubation with an ES-containing bitter fennel infusion was studied in human liver cells. Using a sensitive UHPLC-MS/MS method, we detected E3’-*N*^2^-dG adducts in a multitude of human liver samples. The adduct levels correlated positively with SULT1A1, but not CYP1A2 protein expression. Noteworthy, the E3’-*N*^6^-dA adduct was not found in the analyzed human liver tissue. Further experiments revealed that E3’-*N*^2^-dG adducts accumulate in metabolically competent human liver cells after repetitive short-term exposure to ES, whereas the E3’-*N*^6^-dA adduct was not detected at all. Finally, we provided evidence that ES from a bitter fennel tea preparation induces E3’-*N*^2^-dG adducts in human liver cells, however at lower levels than expected. Altogether, this study demonstrated the occurrence of ES-derived DNA adducts in human liver tissue at levels comparable to the structurally related methyleugenol. Notably, the found DNA adduct levels are well below those reported to cause mutagenicity and clastogenicity in human cells and rodent in vivo models.

**Supplementary Information:**

The online version contains supplementary material available at 10.1007/s00204-026-04364-5.

## Introduction

Phenylpropenes are compounds that occur in various herbs and spices as abundant constituents of the essential oil (Atkinson 2018; Eisenreich et al. 2021). The most important representatives of this chemical class include estragole (ES), methyleugenol (ME), safrole (SA), *trans*-anethol (*t*-AN) and others. Relevant ES sources are the essential oil of bitter fennel (3.5–12%), sweet basil (20–89%), tarragon (60–75%) and star anise (5–6%) (EMA/HMPC 2024). Human exposure to ES is mainly attributable to the diet and herbal medicinal products, resulting in the estimated daily uptake of 0.5–5 mg ES (EMA/HMPC 2024; Punt et al. 2016).

Following its oral uptake and gastrointestinal resorption, ES undergoes extensive hepatic metabolism by phase I and phase II enzymes, which can either result in its detoxification and excretion or in its metabolic activation (Eisenreich et al. 2021). In this pathway, ES is transformed to the proximal carcinogen 1′-hydroxy-ES (1′-OH-ES) catalyzed by CYP1A2 and CYP2A6 (Jeurissen et al. 2007). The phase I metabolite 1′-OH-ES then undergoes sulfate conjugation in a SULT1A1 and SULT1C2-dependent manner, forming 1′-sulfooxy-ES (Honda et al. 2016; Suzuki et al. 2012b). This instable metabolite releases a reactive carbenium ion, which can then covalently bind to cellular macromolecules such as DNA, leading to E3′-*N*^2^-dG as main DNA adduct (Punt et al. 2007; Schulte-Hubbert et al. 2020). The same bioactivation pathway was demonstrated for structurally related phenylpropenes, such as ME (Cartus et al. 2012; Herrmann et al. 2012). ES was shown to be genotoxic in vitro and in vivo, causing the formation of liver tumors in rodents (Drinkwater et al. 1976; Miller et al. 1983). Owing to this experimental evidence, ES was classified as genotoxic carcinogen by the Scientific Committee on Food (SCF) (Scientific Committee on Food 2001). Consistent with this classification, the Committee on Herbal Medicinal Products (HMPC) of the European Medicines Agency (EMA) concluded that ES is a genotoxic carcinogen in rodents (EMA/HMPC 2024). Currently, the EFSA is engaged in preparatory work for the evaluation of the safety of estragole-containing preparations from the fruits of sweet and bitter fennel (Tata et al. 2025).

Up to now, no data on ES-dependent DNA adduct formation in humans is available, which would be important for human risk assessment. Recent in vitro studies performed with ES and 1′-OH-ES provided evidence that the ES-derived DNA damage is only partially repaired and can persist (Schulte-Hubbert et al. 2020; Yang et al. 2020, 2021), which should lead to DNA adduct formation in human liver following dietary exposure. Furthermore, we have recently shown that a threshold level of E3′-*N*^2^-dG DNA adducts is required to trigger clastogenicity and cytotoxicity in human liver cells in culture (Ackermann et al. 2025).

The present work addressed whether (1) E3′-*N*^2^-dG adducts are present in human liver samples, (2) DNA adduct accumulation occurs at physiologically relevant exposure levels in human liver cells and (3) ES from consumption of a bitter fennel infusion can cause E3′-*N*^2^-dG adducts in human liver cells. To this end, a sensitive UHPLC-MS/MS method was used to measure ES-derived DNA adducts in metabolically competent human liver cells and in human liver tissue samples. Furthermore, gene expression levels of CYP1A2 and SULT1A1 were assessed by qPCR and protein levels were measured by SDS-PAGE and western blot analysis. Finally, cytotoxicity was determined using the resazurin reduction assay.

## Material and methods

### Cell culture and tissue samples

HepG2 cells stably transduced with human CYP1A2 (Steinbrecht et al. 2020) were maintained in DMEM high glucose without pyruvate (Thermo Fisher Scientific, Waltham MA, USA) supplemented with 10% fetal calf serum (Pan-Biotech, Aidenbach, Germany) and 1% penicillin/streptomycin (Thermo Fisher Scientific, Waltham MA, USA). Cells were mycoplasma negative, as demonstrated by routine PCR testing using Venor®GeM OneStep (Berlin, Germany). Human liver samples from 20 female and 20 male donors were obtained from BioIVT (Brussels, Belgium). Only donors between 18 and 70 years without primary liver disease (e.g. liver cancer, alcohol-associated liver disease, nonalcoholic fatty liver disease, hepatitis, polycystic liver disease, primary biliary cholangitis, primary sclerosing cholangitis) were selected.

### Chemicals and cell treatments

ES (PhytoLab, Vestenbergsgreuth, Germany), *t*-AN (Sigma-Aldrich, St. Louis MO, USA) and (1R)-(-)-fenchone (Fen) (Sigma-Aldrich, St. Louis MO, USA) were dissolved in dimethylsulfoxide (DMSO) (Fisher Scientific, Hampton NH, USA) at stock concentrations of 2 M. The stock solutions were then further diluted in cell culture medium to reach the final test concentration, containing 0.1% DMSO as solvent at every treatment. The anticancer drug etoposide (Hycultec, Beutelsbach, Germany) and the detergent saponin (Serva, Heidelberg, Germany) were used as positive controls.

### Fennel tea preparation and calculations

The bitter fennel fruits were provided by Martin Bauer GmbH & Co. KG (Vestenbergsgreuth, Germany) and analyzed for putative contaminants such as aflatoxins or pyrrolizidine alkaloids according to Ph. Eur. 11.0. The essential oil contributed to 5.91% of the total, water-free weight of the bitter fennel fruits. The essential oil contained ES (2.9%), *t*-AN (71.7%) and Fen (19.0%) as main constituents as determined by a GC-FID (Martin Bauer, Vestenbergsgreuth, Germany). The bitter fennel fruits were used to prepare a fennel fruit dry extract, for which 17.5 kg fennel fruits were macerated for 3.5 h at 80 °C in twelve times the amount of water (Finzelberg GmbH, Andernach, Germany). The aqueous extract was filtered, resulting in a 14.3% (dry matter) native extract yield. This was dried under vacuum for 24 h with the addition of 50% maltodextrin as stabilizer and grinded to powder. The final dry extract was free from any essential oil residues. Furthermore, the fennel oil was obtained by steam distillation in accordance with the German pharmacopoeia (Finzelberg GmbH, Andernach, Germany).

To calculate a possible concentration, which might be ingested by the consumption of fennel tea, a preliminary experiment coordinated by Dr. Björn Feistel (Finzelberg GmbH, Andernach, Germany) was performed to determine the extraction efficiency. Therefore, a fennel tea was prepared according to (EMA/HMPC 2024) by steeping 1.5 g of whole fennel fruits in 250 mL hot water for 15 min. The residue was then determined by infrared-heating until dryness and subsequent balance of the residue. This led to a final concentration of 1 g dry extract/L water, which was used for subsequent genotoxicity and cytotoxicity testing with the above-mentioned oil-free extract. To consider the maltodextrin content in the prepared fennel dry extract, the double weighing method was used to reach a concentration of 1 g/L native dry extract.

To determine the oil concentrations to be used, calculations were made considering 1 g/L dry extract with a theoretical essential oil content of 5.91%, assuming a 100% extraction efficiency of ES. This would translate into a maximum concentration of 11.47 µM ES in the bitter fennel tea infusion. As it was reported previously that 0.1–12% of the oil was extracted in fennel tea infusions (Zeller and Rychlik 2006), we used bitter fennel oil concentrations corresponding to 0.1–20 µM ES. Furthermore, the main fennel oil constituents *t*-AN and Fen were carried along reflecting their native ratio, resulting in concentrations of 492 µM *t-*AN and 132 µM Fen, respectively.

### DNA isolation, hydrolysis and quantification of DNA adducts by mass spectrometry

6 × 10^6^ HepG2-CYP1A2 cells were grown in 10 cm dishes for 24 h and then treated with either fennel tea dry extract, fennel oil or a combination of both for 24 h. As controls, the oil components ES (20 µM) and *t*-AN (492 µM) as well as the solvent control (1% _dd_H_2_O, 0.2% DMSO) were carried along. Afterwards the cells were harvested, washed with PBS and pelleted by centrifugation. The cell pellets were stored at –20 °C until further processing.

For the repeated exposure experiments with ES, 1 × 10^6^ HepG2-CYP1A2 cells were seeded in 6-cm-dishes and allowed to grow over 72 h to reach 50–60% confluency. The cells were then treated for 2 h with ES (1; 10; 100 µM) or the solvent control DMSO (0.1%). Subsequently, the cell culture medium was aspirated, and cells were left to recover for 22 h in fresh medium. This treatment procedure was repeated for up to 4 times. After each treatment-recovery cycle, the cells were harvested, washed with PBS and stored at –20 °C.

In order to measure ES-derived DNA adducts in human liver samples, 25–30 mg of liver tissue was used. Both cell pellets and liver tissue samples were homogenized in lysis buffer and the DNA was isolated as described previously (Carlsson et al. 2022). The DNA concentration and purity were determined with the NanoDrop ND1000 photometer (Thermo Scientific, Waltham MA, USA). As a next step, 30 µg DNA of each sample was digested overnight at 37 °C with micrococcal nuclease (Worthington, Lakewood NJ, USA) and phosphodiesterase II (Worthington, Lakewood NJ, USA) as reported (Carlsson et al. 2022). Afterwards ^15^*N*_5_-E3′-*N*^2^-dG (2 nM), ^15^*N*_5_-E3′-*N*^6^-dA (0.5 nM) as well as ^15^*N*_5_-dG (20 µM) (Silantes, München, Germany) were added as internal standards. The isotope-labeled internal DNA adduct standards and external DNA adduct standard were synthesized as previously described (Schumacher et al. 2013). Subsequently, the samples were incubated overnight at 37 °C with alkaline phosphatase (Sigma-Aldrich, St. Louis MO, USA), followed by precipitation of remaining proteins with ethanol and centrifugation at 20,800 g. The supernatant was narrowed down in a vacuum centrifuge (Eppendorf concentrator plus, Eppendorf, Hamburg, Germany) at 60 °C and 1400 g for 2–4 h and the resulting pellet was dissolved in 75% methanol. After another centrifugation step, the solution was transferred into glass vials with inserts.

The DNA adducts were measured via UHPLC-MS/MS with a multiple reaction method (MRM) using a UHPLC system (Agilent 1290 infinity; Agilent Technologies, Santa Clara CA, USA) as reported recently (Ackermann et al. 2025). For quantification of the adduct levels, a standard calibration curve for both adducts was carried along ranging from 60–100,000 pM, also containing isotope labeled standards for reference. Furthermore, the dG-content of each sample was determined to normalize the adduct levels to the overall nucleoside content (Carlsson et al. 2022; Stegmuller et al. 2018).

### SDS-PAGE and western blot analysis

To determine the SULT1A1 and CYP1A2 levels in human liver tissue, 10–15 mg of each sample were homogenized in RIPA lysis buffer as reported previously (Seiwert et al. 2017). RIPA buffer consisted of 25 mM Tris–HCl, 150 mM NaCl, 0.1% SDS, 1% sodium deoxycholate, 1% NP-40, 0.2 mM Na_3_VO_4_, 200 mM EDTA, 50 mM NaF, 1 mM PMSF as well as a complete protease inhibitor cocktail (pH 7.4). Each homogenized sample was sonicated 10 × on ice (20% Power, 50% Duty Cycle) using Sonopuls HD 2070/ UW 2070 (BANDELIN electronic GmbH und Co. KG, Berlin, Germany) and subsequently centrifuged at 4 °C for 10 min at 15,000 g. The supernatant was then diluted, and protein content was determined using the Bradford assay. 50 µg of protein was used for the SDS-PAGE and western blot analysis as reported (Fahrer et al. 2013). The successful protein transfer onto a nitrocellulose membrane was confirmed by Ponceau S staining before blocking with 5% nonfat dry milk in Tris-buffered saline with Tween-20 (TBS-T). Subsequently, the membrane was incubated with the respective primary antibodies overnight at 4 °C. The membranes were then washed 3 times for 5 min with TBS-T before incubating with the respective secondary antibody coupled with horseradish peroxidase. After final washing steps in TBS-T, the detection was carried out using the Western Lightning Plus-ECL reagent (Perkin Elmer, Waltham MA, USA) with a c300 chemiluminescence imager (Azure Biosystems, Dublin CA, USA). The used primary and secondary antibodies are detailed in Supplementary Table 1.

### Real-time PCR

Gene expression analysis was essentially performed as described previously (Christmann et al. 2016). To quantify the mRNA expression levels of *CYP1A2* and *SULT1A1*, total RNA from HepG2-CYP1A2 as well as liver tissue samples was isolated using the NucleoSpin® RNA Kit from Macherey–Nagel (Düren, Germany). 0.5 µg RNA was then transcribed into cDNA using the Verso cDNA Kit (Thermo Scientific, Dreieich, Germany) according to the manufacturer’s protocol. PCR was performed using the CFX96 Real-Time PCR Detection System (Biorad, München, Germany) and the GoTaq® qPCR Master Mix (Promega, Madison, USA). Analysis was performed using the CFX Manager™ software. Gene expression levels were normalized to *GAPDH* as well as *ACTB*. The used primers are listed in Supplementary Table 2.

### Detection of cytotoxicity via the resazurin reduction assay

4.5 × 10^4^ HepG2-CYP1A2 cells were seeded in 96-well-plates, grown overnight and treated with either fennel tea dry extract, fennel oil or a combination of both for 24 h. For comparison, the oil components ES (20 µM), *t*-AN (492 µM) and Fen (132 µM) as well as the solvent control (1% ddH2O, 0.2% DMSO) were carried along. Etoposide and saponin served as positive controls. After treatment, medium was discarded and replaced by resazurin reaction solution containing 44 µM resazurin in NaCl/Pi buffer (1.1 mM KH_2_PO_4_, 154 mM NaCl, 3.7 mM Na_2_HPO_4_∙H_2_O) diluted 1:10 in medium without supplements. The cells were then incubated for 1.5 h and fluorescence was measured using a Spark® microplate reader (Tecan, Männedorf, Switzerland) with an excitation at 544 nm and emission at 590 nm. The cell viability was calculated relative to the negative control (set to 100%).

### Statistics

Experiments were performed independently at least three times, except otherwise stated. Results from representative experiments are shown. Values underwent Grubbs’ test to exclude outliers and are displayed as mean + standard error of the mean (SEM) using the GraphPad Prism 10.0 Software (GraphPad Software Inc., Boston, MA 02110, USA). Statistical analysis was performed using two-sided Student’s t-test with Welch correction for cytotoxicity and genotoxicity data, whereas a Pearson correlation test was used for correlation analysis of gene or protein levels with DNA adduct data. Statistical significance was defined as *p* ≤ 0.05.

## Results

### E3’-***N***^2^-dG adducts are found in human liver tissue and correlate with SULT1A1 levels

**Fig. 1:**
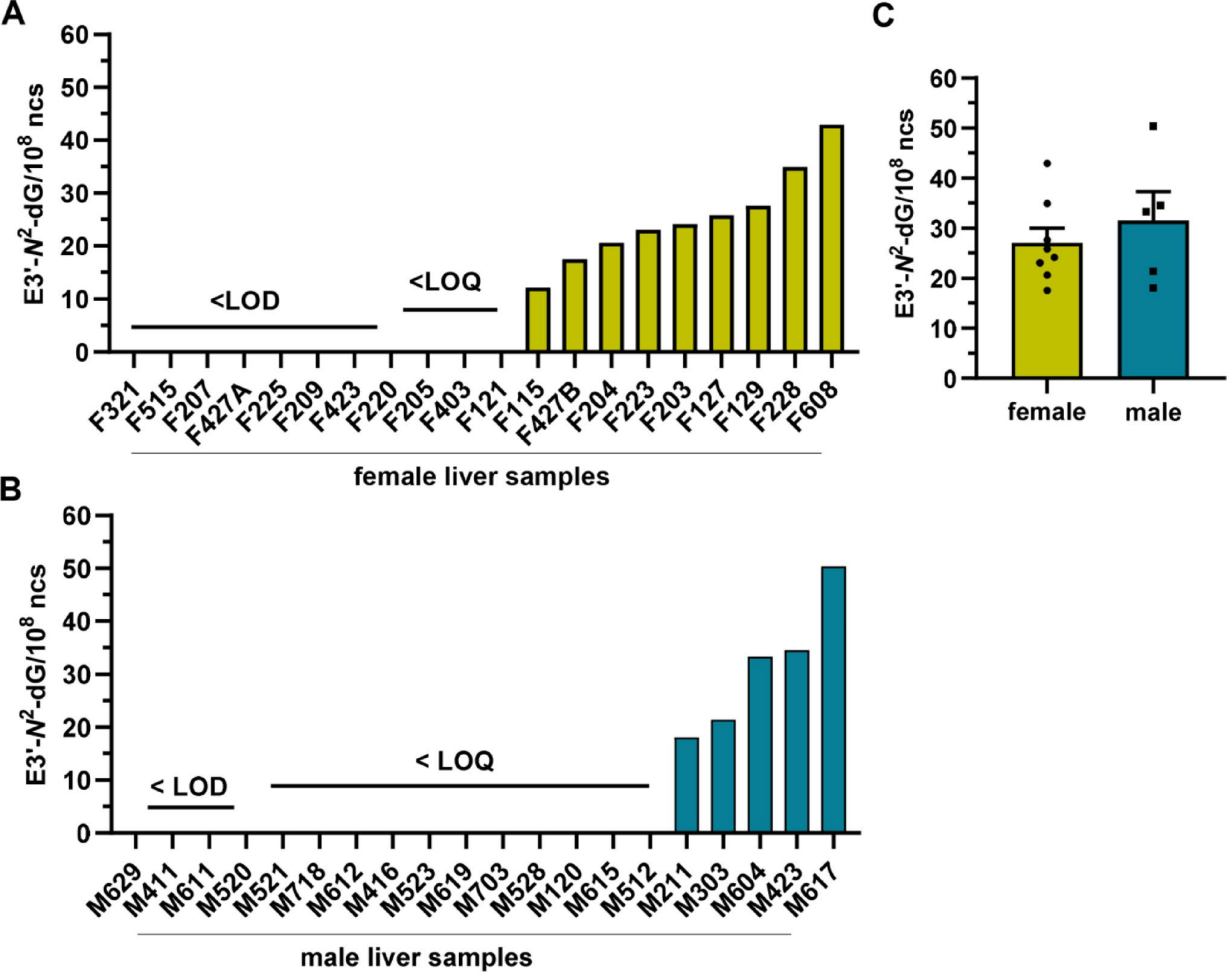
Estragole-derived DNA adducts in human liver biopsies. **A** and **B** Quantification of E3′-*N*^2^-dG adducts in female (**A**) and male (**B**) human liver biopsies (n = 20, each) using UHPLC-MS/MS analysis. LOD: limit of detection; LOQ: limit of quantification. **C** mean E3′-*N*^2^-dG adduct level in female (n = 8) vs. male (n = 5) liver biopsies above the LOQ. Data given as mean + SEM. Statistical analysis was performed using two tailed, unpaired t-test (not significant, *p* > 0.05)

We first analyzed ES-derived DNA adducts in liver tissue samples from 20 female and 20 male human donors using our established UHPLC-MS/MS method (Ackermann et al. 2025). E3’-*N*^2^-dG adducts were detected above the LOD (74.5 pM) in 75% (Fig. 1A and B) and were measured above the LOQ (149 pM) in 35% of all tested liver samples, the latter corresponding to 5 female and 9 male donors (Fig. 1A and B). The male samples showed overall higher adduct levels ranging between 18 and 50 E3’-*N*^2^-dG adducts/10^8^ nucleosides (ncs) with a mean adduct level of 32 adducts/10^8^ ncs (Fig. 1C). The female samples, in turn, ranged between 5.4 and 43 E3’-*N*^2^-dG adducts/10^8^ ncs with a mean adduct level of 26 adducts/10^8^ ncs (Fig. 1C). Noteworthy, the E3’-*N*^6^-dA adduct was not detected at all in the analyzed human liver samples.

**Fig. 2:**
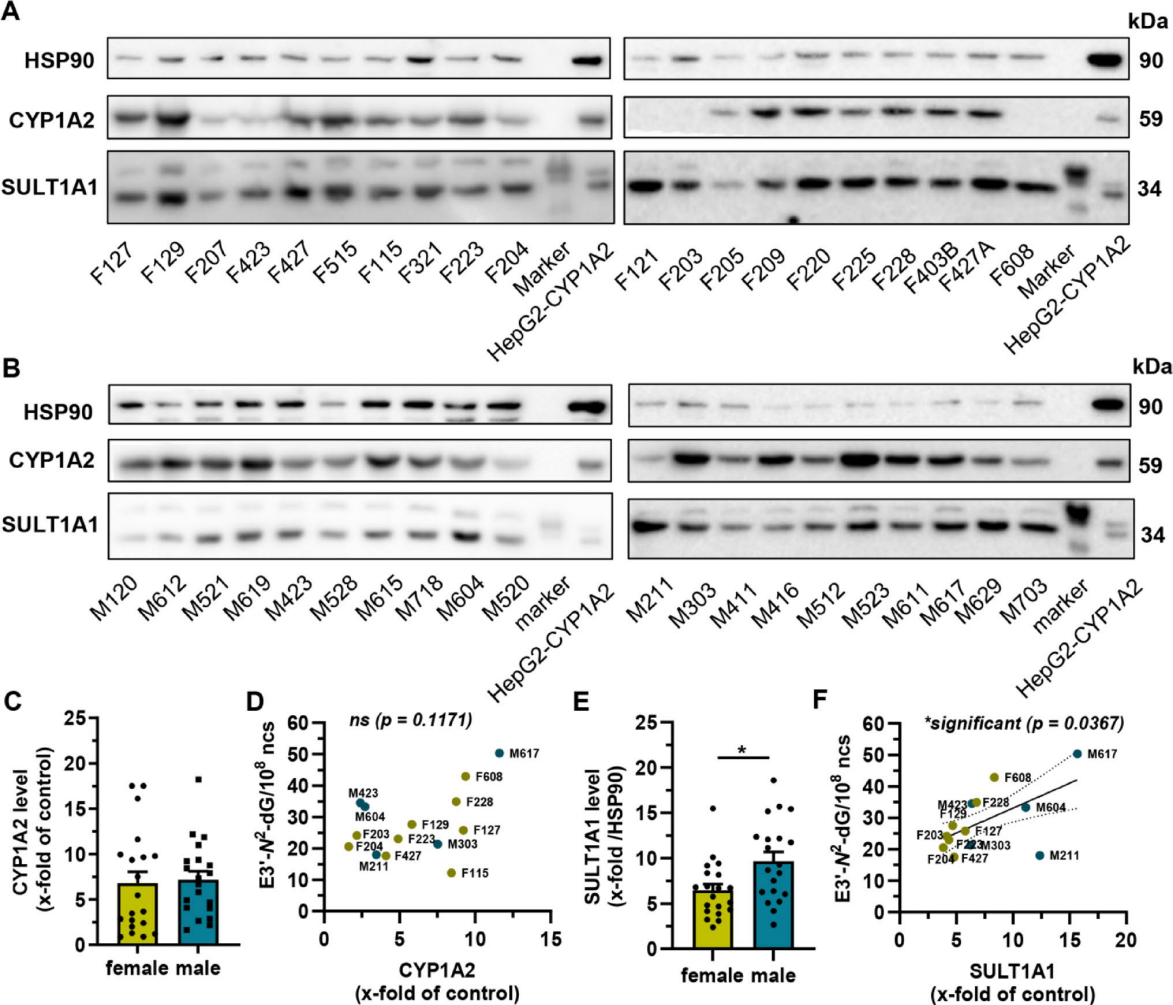
CYP1A2 and SULT1A1 expression in human liver biopsies and correlation with Estragole-derived DNA adduct levels. **A**–**B** Western blot analysis of CYP1A2 and SULT1A1 expression in female (**A**) and male (**B**) human liver biopsies. Hsp90 served as loading control. SULT1A1-expressing HepG2-CYP1A2 cells were used as reference. **C** Relative CYP1A2 levels in female and male liver biopsies (n = 20, each). **D** Correlation analysis of CYP1A2 expression with E3′-*N*^2^-dG adducts in liver samples above the LOQ. **E** Relative SULT1A1 levels in female and male liver biopsies (n = 20, each). **F** Correlation analysis of SULT1A1 expression with E3′-*N*^2^-dG adducts in liver samples above the LOQ. All data given as mean + SEM. Statistical analysis (**C** and **E**) was performed using two tailed, unpaired t-test (not significant, *p* > 0.05; *p ≤ 0.05) and Pearson correlation test (**D** and **F**)

Next, the protein expression levels of CYP1A2 and SULT1A1 as crucial enzymes involved in metabolic activation of ES were analyzed in liver homogenates by SDS-PAGE and western blot detection. Both enzymes were detected in almost all liver samples with varying extent independent of the sex of the donor (Fig. 2A and B; supplementary Fig. 1A and B). As positive control and reference, genetically engineered HepG2-CYP1A2 cells with known SULT1A1 expression were included. The relative mean CYP1A2 levels in liver homogenates from female and male donors were very similar (Fig. 2C). However, no correlation was found between E3’-*N*^2^-dG adduct levels and hepatic CYP1A2 expression (Fig. 2D). The relative SULT1A1 levels were significantly higher in male than in female donors. Interestingly, the E3’-*N*^2^-dG adduct levels correlated significantly with SULT1A1 expression in the human liver tissue samples (Fig. 2F). On the mRNA level, *CYP1A2* showed considerable variability with up to 100-fold differences between the lowest and highest expression (supplementary Fig. 2A). *SULT1A1* expression was found to be more homogeneous across the tested liver samples (supplementary Fig. 2B). However, neither *SULT1A1* nor *CYP1A2* mRNA levels correlated with the measured E3’-*N*^2^-dG adduct levels (supplementary Fig. 2C and D). Taken together, we demonstrated for the first time that significant levels of E3’-*N*^2^-dG adducts occur in human liver tissue, which were positively associated with SULT1A1 protein levels.

### Repetitive exposure of CYP1A2-proficient HepG2 cells leads to accumulation of E3’-***N***^2^-dG adducts

**Fig. 3:**
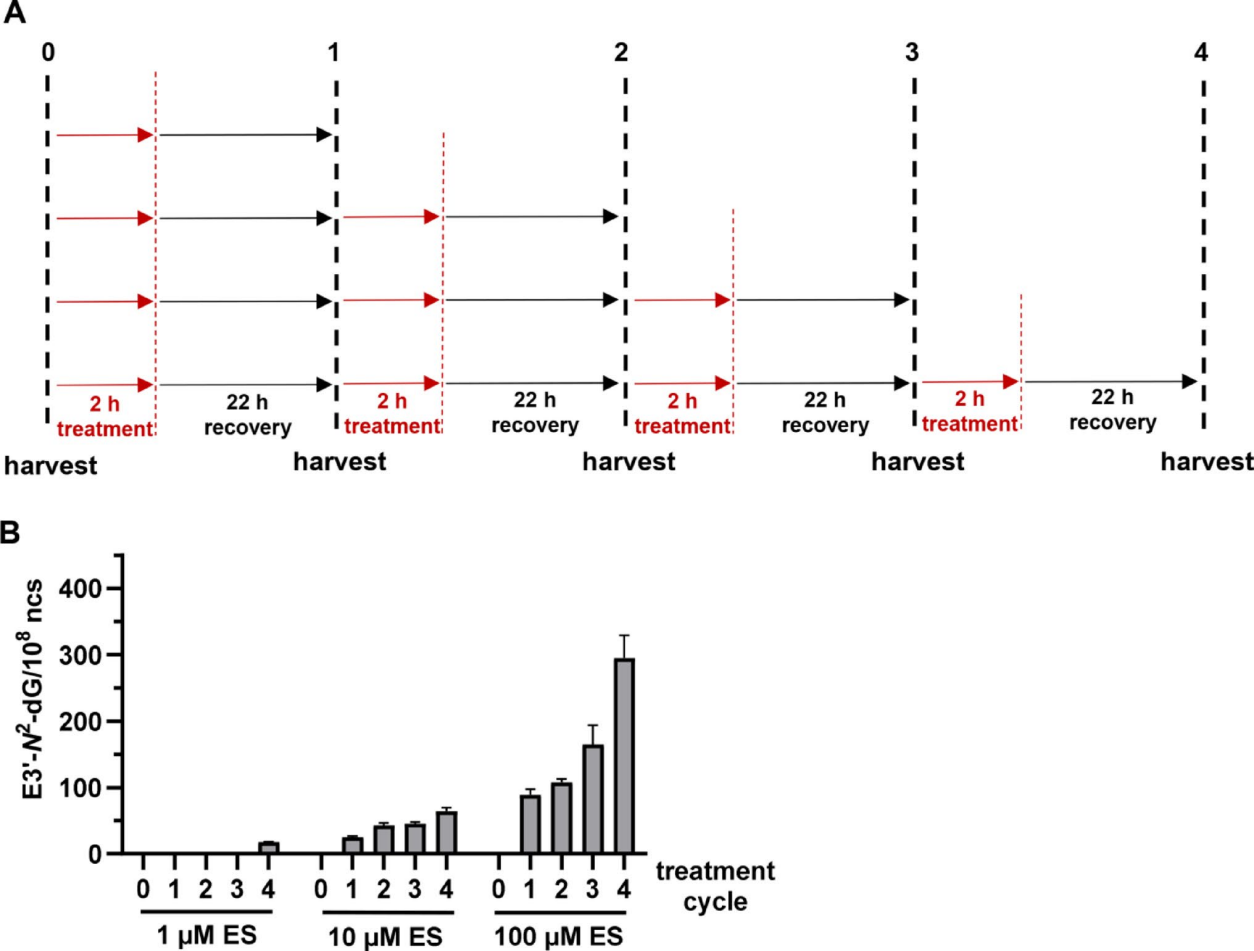
Estragole-derived DNA adduct formation and accumulation in human liver cells. **A** Experimental setup for repetitive exposure of human HepG2-CYP1A2 liver cells to estragole (ES) and DNA adduct kinetics. Cells were treated for 2 h with different ES concentrations (1, 10 and 100 µM) followed by medium exchange and incubation in fresh medium for 22 h (recovery), representing one treatment cycle. This treatment cycle was then repeated three times. After each treatment cycle, cells were harvested and snap-frozen for further DNA adduct analysis. **B** Quantification of E3′-*N*^2^-dG adducts in HepG2-CYP1A2 liver cells after repetitive ES exposure using UHPLC-MS/MS analysis (n = 4). Samples < LOQ are not depicted

In view of these results, we then investigated E3’-*N*^2^-dG adduct formation in human HepG2-CYP1A2 cells after repetitive exposure to ES. To this end, we followed the experimental setup established previously by Yang and colleagues (Yang et al. 2021) with up to 4 exposure cycles consisting of 2 h ES treatment followed by medium exchange and incubation for another 22 h (Fig. 3A). Furthermore, the concentration range was extended to a scenario more relevant for human exposure including 1, 10 and 100 µM ES. Cells were harvested after each treatment cycle and E3’-*N*^2^-dG adducts were determined as mentioned above. At the lowest test concentration (1 µM), E3’-*N*^2^-dG adducts were only measured after day 4, amounting to 18 adducts/10^8^ ncs (Fig. 3B). In contrast to that, the two higher ES concentrations (10 and 100 μM) caused already significant adduct levels after the first treatment cycle with 25 adducts /10^8^ ncs and 89 adducts/10^8^ ncs, respectively (Fig. 3B). These adduct levels increased in a treatment cycle-dependent manner to a maximum of 64 adducts/10^8^ ncs (10 μM) and 296 adducts/10^8^ ncs (100 μM), respectively. Interestingly, E3’-*N*^6^-dA adducts were not detectable in any sample. In summary, these experiments revealed that E3’-*N*^2^-dG adducts accumulate in metabolically competent liver cells after repetitive short-term exposure in a rather sublinear manner.

### A bitter fennel tea preparation gives rise to E3’-***N***^2^-dG adducts in human HepG2-CYP1A2 liver cells

**Fig. 4:**
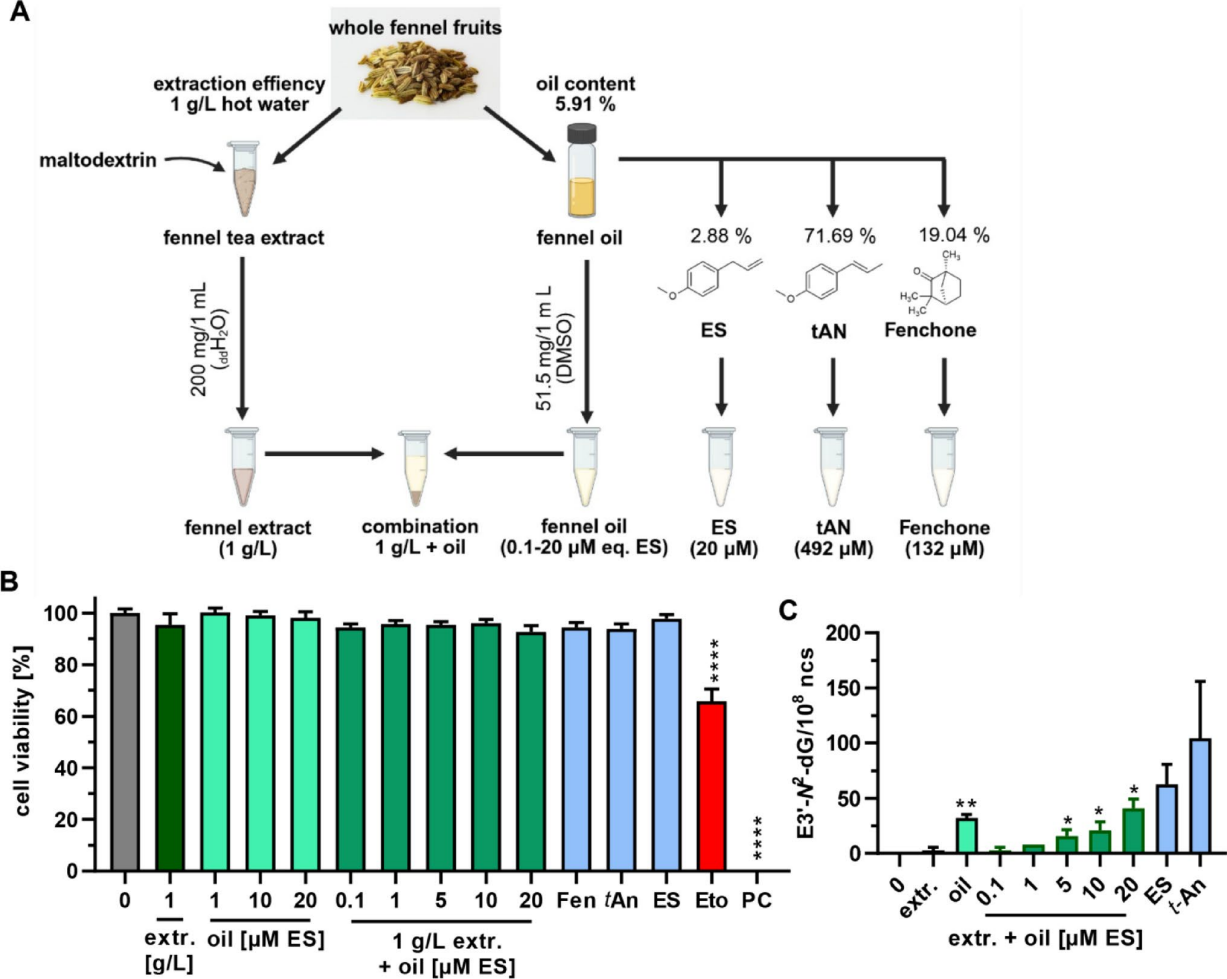
Impact of an estragole-containing fennel tea preparation on DNA adduct formation and viability in human liver cells. **A** Bitter fennel fruits were extracted with hot water, filtered and vacuum-dried together with 50% maltodextrin as stabilizer, resulting in an oil-free fennel tea extract. Furthermore, the fennel oil was obtained by steam distillation of the fennel fruits, consisting of estragole (ES), *trans*-anethole (*t*-AN) and fenchone (Fen). The fennel tea extract was reconstituted in water (1 g/L), while the fennel oil was diluted in DMSO (51.5 mg/mL). Both fractions were then combined, resulting in a reconstituted fennel tea preparation with increasing oil (and thus ES) content. Cartoon created in BioRender. Fahrer, J. (2026) https://BioRender.com/c6e23ip ** B** Impact of fennel tea extract, fennel oil and the combination on the viability of HepG2-CYP1A2 cells after incubation for 24 h. Furthermore, cells were treated with the isolated fennel oil ingredients ES (20 µM), tAN (492 µM) or Fen (132 µM). The cytotoxic anticancer drug etoposide (Eto) was used as positive control. Data are shown as mean + SEM (n = 3–4). Statistical analysis was performed using two tailed, unpaired t-test versus medium control (****p < 0.0001). **C** Quantification of E3′-*N*.^2^-dG adducts in HepG2-CYP1A2 liver cells treated as indicated for 24 h using UHPLC-MS/MS analysis. Data are indicated as mean + SEM (n = 4). Statistical analysis versus control using unpaired t-test with Welch’s correction (*p < 0.05, **p < 0.01)

To detail the human relevance of dietary ES intake, a fennel tea infusion was analyzed regarding its genotoxic and cytotoxic potential, thereby considering possible matrix effects, such as the influence of polyphenols or the mixture toxicity of different phenylpropens found in fennel oil, such as ES and t-AN. To this end, bitter fennel fruits with an essential oil content of 5.91% were used, which comprised 2.9% ES, 19.0% Fen and 71.7% *t*-AN. The bitter fennel fruits were extracted in hot water, followed by the addition of maltodextrin and vacuum drying, resulting in a fennel tea dry extract. Furthermore, the bitter fennel oil was obtained by steam distillation (Fig. 4A). To simulate the consumption of a fennel tea infusion, a concentration of 2 g/L essential-oil-free dry extract (consisting of 50% maltodextrin, resulting in 1 g/L extract concentration) in combination with an increasing essential oil concentration of 5.91–118.2 mg/L was used (Fig. 4A). Those concentrations are roughly equivalent to 0.1–20 μM ES, 0.7–132 μM Fen and 2.5–492 μM *t*-AN. All components were tested separately and as combination of dry extract with varying oil concentrations. First, the putative cytotoxicity was determined after 24 h in HepG2-CYP1A2 cells, which revealed no cytotoxic effect at all (Fig. 4B). Neither the reconstituted essential-oil-free dry extract nor the essential oil or the combination reduced cell viability. The pure substances ES, Fen and *t*-AN were also tested negative, whereas the positive controls etoposide and saponin caused pronounced cytotoxicity (Fig. 4B). Furthermore, the E3’-*N*^2^-dG adducts in genomic DNA were measured by UHPLC-MS/MS, revealing adduct formation upon incubation with the essential oil, extract-essential-oil-combination, ES or *t*-AN (Fig. 4C). Treatment with the essential oil caused 32 adducts/10^8^ ncs, while the combination resulted in a concentration-dependent increase from 8 to 41 adducts/10^8^ ncs. The treatment with 20 μM pure ES (63 adducts/10^8^ ncs) and 492 μM pure *t*-AN (105 adducts/10^8^ ncs) caused 2–threefold higher adduct levels than the extract-essential-oil-combination despite a comparable amount of both phenylpropens. This might be attributable to a competition of ES and *t*-AN for the enzymes involved in metabolic activation. Furthermore, the results indicate that the measured E3’-*N*^2^-dG adducts are predominantly formed from ES, but not from *t*-AN. Collectively, our results showed that ES from dietary sources (here a bitter fennel tea preparation) can induce E3’-*N*^2^-dG adducts in human liver cells but these might be lower than expected.

## Discussion

The present work focused on ES-triggered DNA adduct formation in human liver cells (in vitro) and tissue (in vivo) taking into account repetitive exposure at relevant concentrations from dietary sources. First, our UHPLC-MS/MS measurements revealed ES-derived DNA adducts in a substantial number of the analyzed human tissues samples, ranging between 5 and 50 E3’-*N*^2^-dG adducts/10^8^ ncs. Furthermore, the adduct levels significantly correlated with the hepatic SULT1A1 protein expression. This is in line with a previous study on the structurally related phenylpropene ME. The major ME-derived adduct, *N*^2^-MIE-dG, was also found in a vast majority of human liver samples at levels between 2 and 36 adducts/10^8^ ncs (Herrmann et al. 2013). This was substantiated in a follow-up study with a larger sample size, in which the *N*^2^-MIE-dG adduct number was positively associated with SULT1A1 expression level (Tremmel et al. 2017). Noteworthy, our analysis of ES-derived DNA adducts was performed with liver tissue samples from donors without liver disease. However, no information is available on the dietary habits or the intake of herbal drugs in the liver tissue donors. ES-derived DNA adduct formation was previously demonstrated in the liver tissue of rats, in which single oral administration of 5 mg ES/kg body weight caused 100 – 400 E3’-*N*^2^-dG adducts/10^8^ nts after 48 h (Paini et al. 2012). The measured DNA adduct levels (5—50 E3′-*N*^2^-dG/10^8^ ncs) in human liver tissue are clearly below the adduct levels required to trigger clastogenicity in cultured human liver cells after single ES exposure, which ranged between 240 and 2230 E3′-*N*^2^-dG adducts/10^8^ ncs (Ackermann et al. 2025). It should also be mentioned that *t*-AN could have contributed to the measured E3′-*N*^2^-dG adducts in liver tissue due to the same underlying metabolic activation pathway, thus resulting in common DNA adducts (Bergau et al. 2021). However, the adduct levels induced by *t*-AN were much lower than those caused by equimolar ES concentrations (Bergau et al. 2021), suggesting that *t*-AN is a minor contributor to the global E3′-*N*^2^-dG adduct burden. This is particularly relevant for bitter fennel oil, in which *t*-AN represents the major constituent with a 5 to 14-fold higher abundance compared to ES (Raal et al. 2012).

Next, we demonstrated that the E3’-*N*^2^-dG adduct accumulates in human liver cells after repetitive exposure to ES, which was already detectable at an ES concentration of 1 µM. These data extend the findings obtained in a previous study in HepaRG cells exposed to 50 µM ES using the same experimental setup (Yang et al. 2021). The authors determined an adduct accumulation rate of 17.5 E3’-*N*^2^-dG adducts/10^8^ nts and cycle (Yang et al. 2021), which is similar to the DNA adduct accumulation rate observed herein. The DNA adduct accumulation already at low ES concentrations is very likely attributable to inefficient DNA repair, as suggested by a previous study in DNA repair proficient and deficient CHO cell models (Yang et al. 2020). In line with these findings, persistent levels of DNA damage upon ES treatment were observed in primary rat hepatocytes (Schulte-Hubbert et al. 2020). Obviously, further studies are required to elucidate how E3′-*N*^2^-dG adducts are repaired.

The DNA adduct accumulation upon repetitive ES exposure in human liver cell models is consistent with our finding that ES-derived DNA adducts occur in human liver tissue. This raises the question as to whether the persistent adducts can induce mutations or cause cytotoxicity. During DNA replication, E3′-*N*^2^-dG adducts may block the replicative DNA polymerases δ and ε, giving rise to replication stress. Interestingly, this has been shown for ME-derived DNA damage, which reduced DNA replication speed and caused an ATR-mediated DNA damage response (Carlsson et al. 2022). Interestingly, both E3′-*N*^2^-dG and *N*^2^-MIE-dG adducts were shown to be substrates for the DNA translesion polymerases κ and η, which replicate across both adducts in an error-free manner in vitro (Deshmukh et al. 2023). On the one hand, this very likely explains the rather low acute toxicity of ES and ME, since DNA replication can proceed via the TLS pathway. On the other hand, the reported error-free replication suggests a limited mutagenic potential. This view is supported by mutagenicity studies in F344 gpt delta rats and gpt delta mice, which showed significant levels of mutagenicity only at a dose of 75 mg ES/kg bw or higher (Suzuki et al. 2012a, 2012b). In contrast, no significant increase in mutation frequency was observed at 37.5 or 22 mg/kg bw despite the presence of ES-derived DNA adducts (Suzuki et al. 2012a, 2012b).

Finally, we addressed the question whether exposure to ES via a bitter fennel preparation can cause DNA adducts in human liver cells. Our experiments showed the formation of E3′-*N*^2^-dG adducts both by the essential bitter fennel oil as well as the combination of bitter fennel oil and an aqueous extract. However, the measured levels were lower as originally anticipated based on calculations and comparison to the pure substances, *i.e.,* ES and *t*-AN. One explanation may be the competition of both ES and *t*-AN for CYP-mediated bioactivation to the resulting 1’-OH-ES and 3’-OH-*t*-AN, respectively. A previous in vitro study provided evidence that 1′-hydroxylation of ES is significantly inhibited in the presence of ME due to the competition for CYP1A2 (Jeurissen et al. 2007). This might also be conceivable for the co-incubation of ES and *t*-AN, thus leading to a decreased overall DNA adduct formation. Furthermore, matrix effects have to be considered in bitter fennel preparations. The polyphenol nevadasine, an ingredient of basil extract, was shown to inhibit ES-dependent DNA adduct formation in liver cells and in vivo in Sprague–Dawley rats, which was attributed to SULT inhibition (Alhusainy et al. 2013; Jeurissen et al. 2008). However, a PBK-model based prediction suggested that this inhibitory effect is unlikely to occur under realistic exposure scenarios in humans (Rietjens et al. 2015).

Altogether, this study showed for the first time that ES-derived DNA adducts are detected in human liver tissue at levels comparable to the structurally related ME, thereby reflecting chronic human exposure. Furthermore, our study demonstrates that ES from dietary sources (e.g., bitter fennel infusion) can cause DNA adducts and repetitive exposure results in DNA adduct accumulation, although at very low velocity. Importantly, the DNA adduct burden measured in human liver tissue is below the DNA adduct level that gives rise to mutagenicity and clastogenicity in cells and rodent models. However, further research is warranted regarding the fate of ES-derived DNA adducts as well as mixture effects of ES and structurally related phenylpropenes such as ME and *t*-AN.

## Supplementary Information

Below is the link to the electronic supplementary material.

Supplementary Material 1

## Data Availability

All datasets generated and analyzed during this study were included in the manuscript and the supplementary information. They are also available from the corresponding author upon reasonable request.
