# Supplementary material for: Formation and persistence of estragole-derived DNA adducts in human liver cells and tissue
Source: Arch Toxicol. 2026 Mar 31;100(7):3173–83. doi: 10.1007/s00204-026-04364-5 (PMC13309446; doi:10.1007/s00204-026-04364-5)
Supplement: Supplementary file 1 — Supplementary Material 1 [file 204_2026_4364_MOESM1_ESM.docx]

**Supplementary Figures**

**Figure 1**

**
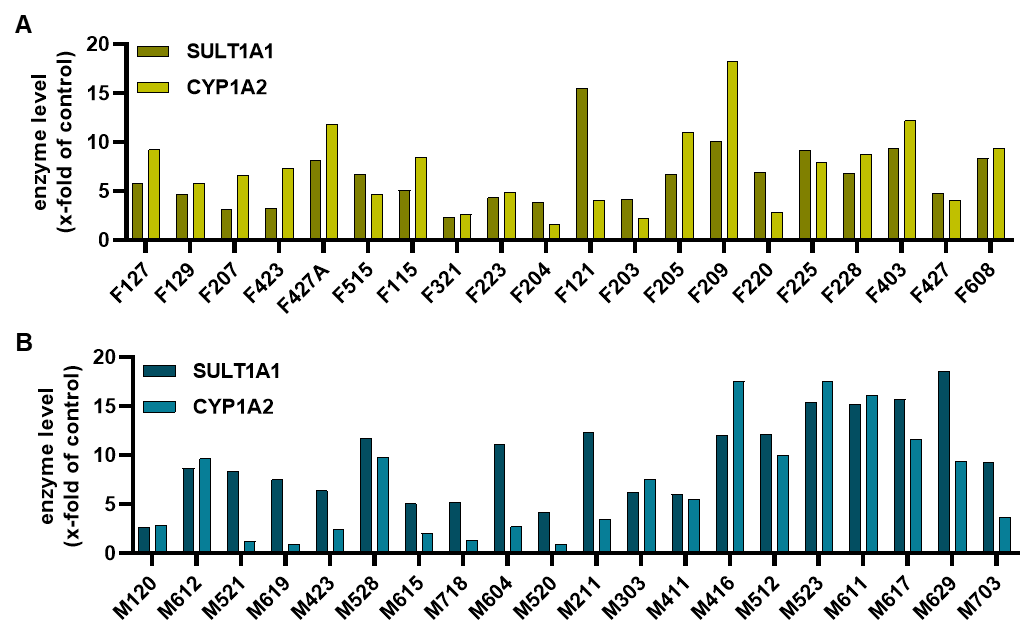
**

**Fig. 1: CYP1A2 and SULT1A1 protein expression in human liver biopsies.** Evaluation of western blot analysis for CYP1A2 and SULT1A1 expression in female (A) and male (B) human liver biopsies normalized to HepG2 cells with CYP1A2 and SULT1A1 expression. Hsp90 expression served as loading control.

**
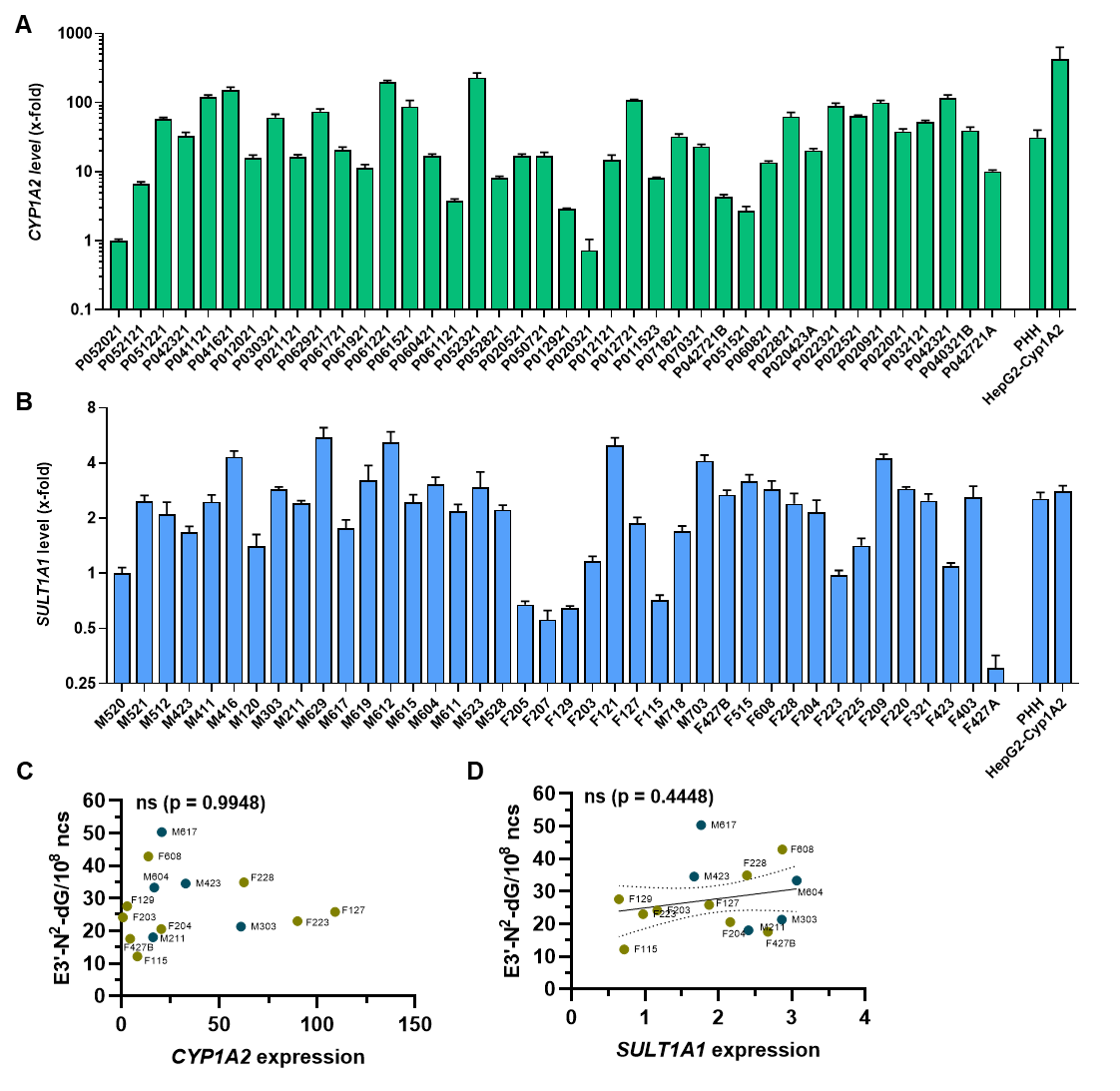
Figure 2**

**Fig. 2: *CYP1A2* and *SULT1A1* expression in human liver biopsies and correlation with estragole-derived DNA adducts. A - B** Gene expression analysis of *CYP1A2* (A) and *SULT1A1* (B) expression in female and male human liver biopsies using qPCR. Gene expression levels were normalized to *ACTB* and *GAPDH* expression as housekeeping genes. **C** Correlation analysis of *CYP1A2* expression with E3′-*N*^2^-dG adducts in liver samples above the LOQ. **D** Correlation analysis of *SULT1A1* expression with E3′-*N*^2^-dG adducts in liver samples above the LOQ. Data given as mean + SD. Statistical analysis (C and D) was performed using two tailed, unpaired Pearson correlation test.

**Supplementary Tables**

**Table 1: Antibodies used in this study.**

| **Antibody** | **Catalog No.** | **Provider** |
| --- | --- | --- |
| Anti-Hsp90α/β (F-8), mouse monoclonal | sc-13119 | Santa Cruz Biotechnology,  Heidelberg, Germany |
| Anti-CYP1A2 (D15), mouse monoclonal | sc-53241 | Santa Cruz Biotechnology,  Heidelberg, Germany |
| Anti-SULT1A1, polyclonal rabbit | GTX55811 | Genetex, Irvine, California, USA |
| mouse IgGκ binding protein-HRP | sc-516102 | Santa Cruz Biotechnology,  Heidelberg, Germany |
| Goat-anti-Rabbit-HRP | #7074 | Cell Signaling Technology,  Danvers, Massachusetts, USA |

**Table 2: Used primer sequences to determine *CYP1A2* and *SULT1A1* expression levels by qPCR**

| **qPCR-Primer** | **Sequence (5‘-3‘)** |
| --- | --- |
| *hCYP1A2-for* | TTCGTAAACCAGTGGCAGGT |
| *hCYP1A2-rev* | AGGGCTTGTTAATGGCAGTG |
| *hSULT1A1-for* | CAAAGGATGTGGCAGTTTCC |
| *hSULT1A1-rev* | CCGACCATGAACTTCTCCAG |
| *hACTB-for* | tggcatccacgaaactacc |
| *hACTB-rev* | gtgttggcgtacaggtctt |
| *hGAPDH-for* | catgagaagtatgacaacag |
| *hGAPDH-rev* | atgagtccttccacgata |
